# Postoperative inflammation and insulin resistance in relation to body composition, adiposity and carbohydrate treatment: A randomised controlled study^☆^

**DOI:** 10.1016/j.clnu.2018.01.032

**Published:** 2019-02

**Authors:** Nilanjana Tewari, Sherif Awad, František Duška, Julian P. Williams, Andrew Bennett, Ian A. Macdonald, Dileep N. Lobo

**Affiliations:** a Gastrointestinal Surgery, Nottingham Digestive Diseases Centre, National Institute for Health Research (NIHR) Nottingham Biomedical Research Centre, Nottingham University Hospitals NHS Trust and University of Nottingham, Queen's Medical Centre, Nottingham NG7 2UH, UK; b The East-Midlands Bariatric and Metabolic Institute (EMBMI), Derby Teaching Hospitals NHS Foundation Trust, Royal Derby Hospital, Derby DE22 3NE, UK; c Department of Anaesthesia and Intensive Care, Kralovske Vinohrady University Hospital and The Third Faculty of Medicine, Prague, Czech Republic; d Department of Critical Care, Nottingham University Hospitals NHS Trust, Queen's Medical Centre, Nottingham NG7 2UH, UK; e FRAME Laboratory, School of Life Sciences, University of Nottingham, Queen's Medical Centre, Nottingham NG7 2UH, UK; f School of Life Sciences, University of Nottingham, Queen's Medical Centre, Nottingham NG7 2UH, UK; g MRC/ARUK Centre for Musculoskeletal Ageing Research, University of Nottingham, Queen's Medical Centre, Nottingham NG7 2UH, UK

**Keywords:** Metabolic response, Carbohydrate, Obesity, Insulin resistance, Abdominal surgery, Inflammation

## Abstract

**Background & aims:**

The aims of this study were to identify whether differences in distribution of adipose tissue and skeletal muscle in obese and non-obese individuals contribute to the magnitude of the postoperative inflammatory response and insulin resistance, with and without preoperative treatment with carbohydrate drinks.

**Methods:**

Thirty-two adults (16 obese/16 non-obese) undergoing elective major open abdominal surgery participated in this 2 × 2 factorial, randomised, double-blind, placebo-controlled study. Participants received Nutricia preOp^®^ or placebo (800 ml on the night before surgery/400 ml 2–3 h preoperatively) after stratifying for obesity. Insulin sensitivity was measured using the hyperinsulinaemic-euglycaemic clamp preoperatively and on the 1st postoperative day. Vastus lateralis, omental and subcutaneous fat biopsies were taken pre- and postoperatively and analysed after RNA extraction. The primary endpoint was within subject differences in insulin sensitivity.

**Results:**

Major abdominal surgery was associated with a 42% reduction in insulin sensitivity from mean(SD) M value of 37.3(11.8) μmol kg^−1^ fat free mass (FFM) to 21.7(7.4) μmol kg^−1^ FFM, but this was not influenced by obesity or preoperative carbohydrate treatment. Activation of the triggering receptor expressed on myeloid cells (TREM1) pathway was seen in response to surgery in omental fat samples. In postoperative muscle samples, gene expression differences indicated activation of the peroxisome proliferator-activated receptor (PPAR-α)/retinoid X-receptor (RXR-α) pathway in obese but not in non-obese participants. There were no significant changes in gene expression pathways associated with carbohydrate treatment.

**Conclusion:**

The reduction in insulin sensitivity associated with major abdominal surgery was confirmed but there were no differences associated with preoperative carbohydrates or obesity.

## 1 Introduction

Major surgery induces inflammation and metabolic stress, processes which may be exacerbated by pre- and postoperative starvation, leading to postoperative insulin resistance (IR) [1], [2], [3], [4], [5]. IR is a state in which, under hyperinsulinaemic-euglycaemic conditions, glucose uptake is below the lowest quartile for the population under investigation [6], and obese individuals may be more prone to develop IR [5].

As IR is associated with an increase in postoperative complications [1], [2], [4], [5], measures to attenuate it may be beneficial. Some studies have suggested that administration of carbohydrate polymer (e.g. maltodextrin) drinks up to 2 h preoperatively may reduce postoperative IR [7], [8], [9]. However, three meta-analyses have not shown a reduction in complications or overall hospital stay when patients receiving carbohydrate treatment were compared with those receiving placebo or no treatment [10], [11], [12]. Nevertheless, a one-day reduction in hospital stay was seen in patients undergoing major abdominal surgery after carbohydrate treatment [11].

The aims of this study were to identify whether differences in the distribution and physiology of adipose tissue and skeletal muscle in obese and non-obese individuals contribute to variation in the magnitude of the postoperative inflammatory response and IR, with and without preoperative carbohydrate treatment.

## 2 Methods

### 2.1 Study design, ethics, trial registration and inclusion and exclusion criteria

This 2 × 2 factorial, randomised, double-blind, placebo-controlled study was set in a university teaching hospital. Adults aged 18–80 years undergoing elective major open abdominal surgery were enrolled after providing informed written consent. The National Research Ethics Service approved the study (11/EM/0232), which was registered at www.controlled-trials.com (ISRCTN16597586). Exclusion criteria were pregnancy, >10% weight loss in the preceding 3 months, metabolic and endocrine disorders including diabetes mellitus and steroid usage, known gastro-oesophageal reflux disease or hiatus hernia, history of pulmonary aspiration, and suspicion of alcohol or drug abuse. Participants were classified as obese (BMI ≥30 kg m^−2^ or waist circumference ≥94 cm in men or ≥80 cm in women) (n = 16) or non-obese (n = 16).

### 2.2 Interventions, randomisation and blinding

Central computerised randomisation was performed for participants to receive Nutricia preOp^®^ (Nutricia Clinical Care, Trowbridge, UK) or placebo (Tovali Sugar Free Whole Lemon Drink diluted 1:4 with water, Tovali Ltd., Carmarthen, UK) (Table 1) after stratifying for obesity. The drinks were prepared in identical opaque bottles by a person not involved in the research. Investigators remained blinded until all analyses were completed.

**Table 1:** Composition of study drinks.

Constituents (per 100 ml)	Unit	Nutricia preOp^®^	Placebo
Energy	kcal kJ	50 215	1.6 7
Protein	g	0	0.02
Carbohydrate	g	12.6	0.04
Maltodextrin	g	10	0
Sugars	g	2.1	0.04
Fat	g	0	Trace
Sodium	mg (mmol)	50 (2.2)	20 (0.9)
Potassium	mg (mmol)	122 (3.1)	Unavailable
Chloride	mg (mmol)	6 (0.2)	Unavailable
Calcium	mg (mmol)	6 (0.1)	Unavailable
Phosphorus	mg (mmol)	1 (0.0)	Unavailable
Magnesium	mg (mmol)	1 (0.0)	Unavailable
Water	g	92	80

### 2.3 End points and sample size

The primary endpoint was within subject differences in perioperative insulin sensitivity (as measured by the hyperinsulinaemic-euglycaemic clamp [HEC] [13]) among obese, non-obese, carbohydrate treated and placebo treated participants. Secondary endpoints were correlation between body composition and changes in IR, differences in inflammatory cytokine gene expression, and differences in muscle and fat genes controlling carbohydrate and fat oxidation and insulin signalling. Complications within 30 days of the operation were recorded according to Clavien-Dindo classification [14].

Based on a previous study [7] and with an estimated standard deviation of 10% in measurements of insulin sensitivity, 8 participants were required to detect pair-wise differences of 27% between the two 2 groups (carbohydrate *versus* placebo) with a power of 80% at a significance level of 0.05. Therefore, 8 participants were recruited to each of the four arms.

### 2.4 Experimental methods

Participants had a preoperative screening visit (P1) in the week before surgery. Height, weight, and hip and waist circumferences were measured, and dual X-ray absorptiometry (DXA) scan and HEC performed. Participants were randomised to receive Nutricia preOp^®^ containing 50 g maltodextrin in 400 ml or placebo and consumed 800 ml on the evening before and 400 ml 2–3 h before anaesthesia (Table 1). A second HEC was performed on the first postoperative day (P2) (Table 2).

**Table 2:** Time points for blood sampling.

P1	S1	S2	P2
Before surgery (at commencement of preoperative clamp)	Day of surgery, before knife to skin	Day of surgery, after closure of the abdomen	Post-operative day 1 (at commencement of postoperative clamp)

#### 2.4.1 Hyperinsulinaemic-euglycaemic clamp (HEC) [13].

After an overnight fast, two intravenous cannulae were placed, one in an antecubital vein for the infusion of insulin (Actrapid, Novo Nordisk, Denmark) and 10% glucose, and the other retrograde into a dorsal hand vein which was kept in a hot box for sampling of arterialised blood. A baseline blood sample for measurement of fasting blood glucose, and inflammatory and hormonal markers was taken. After a 10-min priming infusion, insulin infusion (1 unit ml^−1^ 0.9% saline) was held constant at 120 mIU m^−2^ min^−1^ for 110 min. Blood glucose concentration was determined every 5 min using the YSI 2300 Stat Plus (YSI Inc., Yellow Springs, OH). Blood glucose concentration was clamped at 4.5 mmol l^−1^ by infusion of variable amounts of glucose. The total body glucose disposal rate (M-value) was calculated during the final 15 min (steady-state) and was used as a measure of insulin sensitivity [13]. Insulin concentrations were measured during the clamp to allow calculation of M/I. (Supplementary Digital Content Fig. 1).

#### 2.4.2 Body composition analysis

One CT image slice for each participant at the third lumbar vertebra level was selected and the images were analysed using SliceOmatic V4.2 software (Tomovision, Montreal, Canada) to quantify fat free mass (FFM), fat mass (FM), skeletal muscle index (SMI) and myosteatosis (Supplemental Digital Content – Experimental) [15], [16], [17], [18], [19], [20].

#### 2.4.3 Collection and analysis of blood samples

The concentrations of tumour necrosis factor-α (TNFα), interleukin-6 (IL-6), C-reactive protein (CRP), triacylglycerol (TAG), blood glucose, plasma cortisol, serum insulin, serum non-esterified fatty acids (NEFA) and free fatty acids (FFA) were measured at four time points (Table 2). Assays are described in the Supplemental Digital Content – Experimental. The TNF assay was not highly sensitive, with detection limit of 1 pg ml^−1^.

#### 2.4.4 Skeletal muscle and subcutaneous adipose tissue biopsies

Vastus lateralis (VL) muscle biopsies were obtained using a Weil-Blakesley forceps (medizintechnik, Stuttgart, Germany). Subcutaneous adipose tissue and omental tissue were also collected. The samples were snap frozen in liquid nitrogen and stored at −80 °C.

#### 2.4.5 RNA extraction

Biopsy samples were defrosted and total RNA was isolated using Tri reagent according to the manufacturer's instructions. The RNA pellet was air-dried for 10 min and then resuspended in 50 μL of RNase free water, and cDNA was prepared and quantified (Supplemental Digital Content – Experimental).

#### 2.4.6 TaqMan low density gene array analysis

A custom TaqMan^®^ Array Micro Fluidic Card was designed for each tissue for gene array analysis (Supplemental Digital Content – Experimental and Supplemental Digital Content Tables 1 and 2).

### 2.5 Statistical analyses

Statistical analysis was performed with SPSS^®^ for Windows™ v22 software (IBM, Armonk, NY) and GraphPad Prism v6.04 (GraphPad Software Inc., La Jolla, CA). Data are presented as mean (SE or SD) or median (IQR). The paired and independent samples *t*-tests were used for parametric paired and unpaired data, respectively. The Wilcoxon signed rank and Mann–Whitney U tests were used for non-parametric paired and unpaired data, respectively. The Chi square test was used for categorical data.

Comparisons between the study time points for parametric and non-parametric data were made using the repeated measures ANOVA or the Kruskal–Wallis tests, respectively. Two-tailed p values, with Welch's correction, are reported and differences were considered significant at p < 0.05.

## 3 Results

**Fig. 1:**
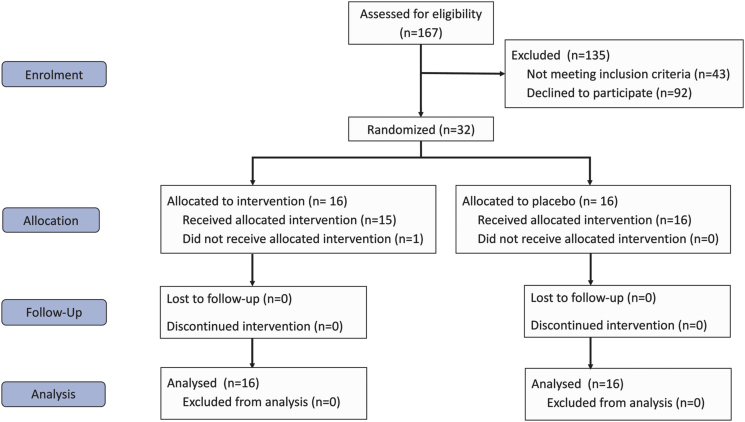
CONSORT flow diagram.

The demographics of the 32 participants (Fig. 1) are shown in Table 3 and surgical procedures are listed in Supplemental Digital Content Table 3.

**Table 3:** Participant demographics.

Parameter	Total (n = 32)	Obese (n = 16)	Non-obese (n = 16)	Carbohydrate treated (n = 16)	Placebo treated (n = 16)	Obese + carbohydrate treated (n = 8)	Obese + placebo treated (n = 8)	Non-obese + carbohydrate treated (n = 8)	Non-obese + placebo treated (n = 8)
Age [median (IQR)] yrs	61 (50–67)	61 (58–66)	63 (46–68)	62 (46–66)	61 (51–68)	60 (44–64)	63 (52–68)	65 (51–72)	61 (51–65)
Gender (M:F)	22:10	12: 4	10: 6	10:6	12:4	5:3	7:1	5:3	5:3
BMI [mean (SD)] kg m^−2^	28.3 (5.5)	31.0 (6.1)	25.7 (3.0)	29.6 (6.3)	27 (4.3)	30.3 (8.3)	28.9 (3.8)	27.5 (5.0)	26.4 (3.6)
Waist circumference [median (IQR)] cm	93 (86.5–106.5)	106.5 (98.6–113)	87.5 (79–92.4)	95 (86.3–110.8)	92.9 (85.8–105.8)	98.6 (92–107.5)	91.5 (74.6–106.5)	90.5 (82.5–112)	92.9 (90–99.6)
Operating time (median, IQR) min	180 (128–240)	160 (128–246)	182 (133–222.5)	152.5 (121–199.5)	188 (152–300)	126 (115.5–182.5)	196.5 (171.5–306.5)	168 (143–223)	160 (120–300)
Blood loss [mean (SD)] ml	542 (465)	631 (533)	469 (405)	570 (216.5–920.5)	400 (108–695)	701 (735)	528 (265)	535 (341)	429 (548)

### 3.1 Hyperinsulinaemic-euglycaemic clamps

There was a 42% decrease (p < 0.001) in insulin sensitivity (Table 4) from M value (mean (SD) of 37.3 (11.8) μmol kg^−1^ FFM to 21.7 (7.4) μmol kg^−1^ FFM, in both obese and non-obese subjects between the preoperative visit and the first postoperative day. However, there was no difference in the magnitude of change in insulin sensitivity between obese and non-obese participants or in those who received and did not receive carbohydrate drinks (Table 4). Plasma insulin concentrations were measured and summarised in Supplemental Digital Content Fig. 1. As the magnitude of change in insulin sensitivity was equivalent when comparing M values pre- and postoperatively or M/I, M values were used for all analyses.

**Table 4:** M values at visit 1 and 2 (mean (SD)) and postoperative reduction insulin sensitivity in obese *vs*. non-obese participants and those receiving carbohydrate *vs*. placebo. There was no influence of gender, age, cancer status or length of surgical procedure on the change in insulin sensitivity (data not shown). The reduction in insulin sensitivity between the preoperative (100%) and postoperative M values was statistically significant (p < 0.001) for all groups.

Groups	All	Obese	Non-obese	Difference between groups
All	(n = 32)	(n = 16)	(n = 16)	p = 0.38
Preoperative M value mean (SD) μmol kg^−1^	37.3 (11.8)	41.6 (11.7)	33.1 (10.7)	
Postoperative M value mean (SD) μmol kg^−1^	21.7 (7.4)	24.5 (7.6)	19.2 (7.4)	
Reduction in insulin sensitivity	42%	41%	42%	
Carbohydrate treated	(n = 16)	(n = 8)	(n = 8)	p = 0.19
Preoperative M value mean (SD) μmol kg^−1^	36.2 (11.4)	32.5 (8.8)	40.3 (13.0)	
Postoperative M value mean (SD) μmol kg^−1^	19.5 (6.1)	18.2 (6.3)	21 (6.0)	
Reduction in insulin sensitivity	46%	44%	48%	
Placebo	(n = 16)	(n = 8)	(n = 8)	p = 0.06
Preoperative M value mean (SD) μmol kg^−1^	38.5 (12.6)	33.8 (13.3)	43.1 (10.8)	
Postoperative M value mean (SD) μmol kg^−1^	22 (8.5)	17.6 (8.4)	26.7 (8.9)	
Reduction in insulin sensitivity	43%	48%	38%	
Difference between groups	p = 0.67	p = 0.23	p = 0.23	

### 3.2 Body composition and insulin sensitivity

There was some correlation between DXA and CT (Supplemental Digital Content Table 4) for both fat mass (r^2^ = 0.486, p < 0.001) and fat free mass (r^2^ = 0.658, p < 0.001). There was no correlation between the presence of sarcopenia and preoperative (r^2^ = 0.233, p = 0.594) or postoperative (r^2^ = 0.318, p = 0.120) IR. Myosteatosis was present in 29.6% participants who had preoperative CT scans. Although there was no correlation between the presence of myosteatosis and sarcopenia (p = 0.152), there was significant correlation between presence of myosteatosis and postoperative IR (r^2^ = 0.746, p = 0.012).

### 3.3 Complications

There was no significant difference in 30-day postoperative complications between obese and non-obese patents and those receiving carbohydrate treatment or placebo, when graded according to the Clavien–Dindo classification [14]. Of the 16 obese patients, 9 had no complications, 3 had Grade I and 4 Grade II complications. Of the 16 non-obese patients, 10 had no complications, 3 Grade I, 2 Grade II and 1 Grade IIIb complications (p = 0.633, Chi-square test). Of the 16 patients who received preoperative carbohydrates, 13 had no complications, 2 Grade I, and 1 Grade II complications. Of the 16 patients receiving placebo, 6 had no complications, 4 Grade I, 5 Grade II and 1 Grade IIIb complications (p = 0.075, Chi-square test). There was no 30-day or in-hospital mortality.

### 3.4 Metabolic data

**Fig. 2:**
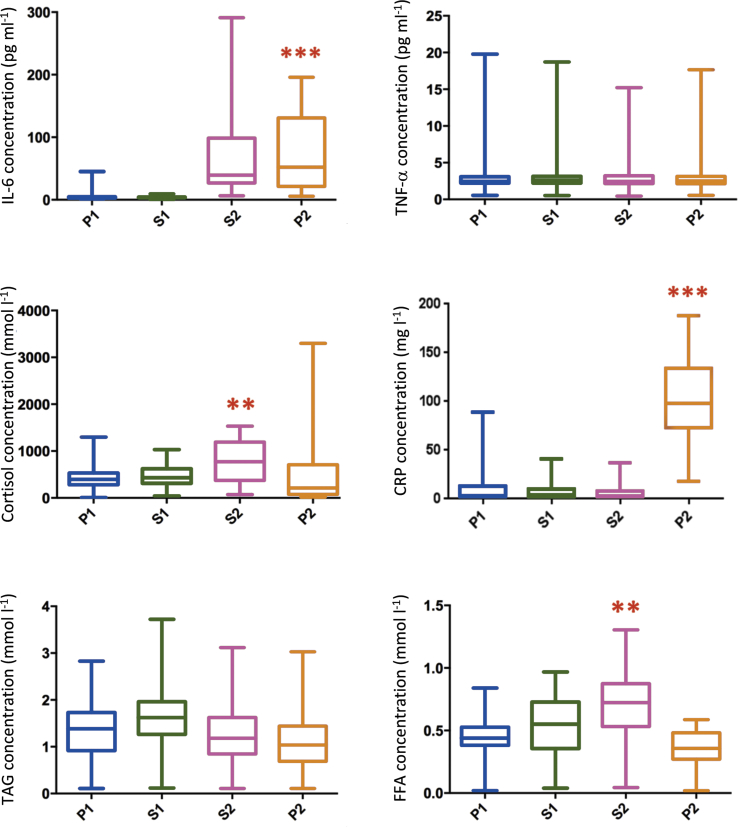
Perioperative changes in concentrations of interleukin 6 (IL-6), tumour necrosis factor α (TNF-α), cortisol, C-reactive protein (CRP), triacylglycerol (TAG) and free fatty acid (FFA). P1 = preoperative, S1 = start of surgery, S2 = end of surgery and P2 = first postoperative day. **p < 0.05, ***p < 0.001.

Concentrations of FFA, TAG, Cortisol and cytokines are shown in Fig. 2. There was a significant increase in IL-6 concentration from the preoperative time point to the end of surgery (p < 0.0001) and there was a significant increase in CRP between the end of surgery and the first postoperative day (p < 0.001). There was no significant difference between obese and non-obese participants in IL-6 (p = 0.203), CRP (p = 0.645), FFA (p = 0.446), cortisol (p = 0.322) or TNFα (p = 0.101) concentrations (data in figure – combined placebo and carbohydrate treated data as there were no differences between groups).

### 3.5 Gene expression analysis of muscle and fat biopsies

Taqman low density arrays were designed to contain assays for a selection of genes involved in carbohydrate and lipid metabolism, insulin signalling, muscle atrophy and inflammatory pathways.

#### 3.5.1 Effects of surgery

Omental fat sample gene expression results indicated an elevation of inflammatory pathway genes in response to surgery. Database for annotation visualisation and integrated discovery (DAVID) analysis of omental fat data revealed that the top two enriched Kyoto encyclopaedia of genes and genomes (KEGG) pathways highlighted were ‘regulation of cytokine production’ (NLRP3, IRAK3, IL1B, IL18, C3, HIF1A, CASP1, TGFB1, ADIPOQ, MYD88, IL6, PPARG, IL10, TNF, SOD1, CIDEA; p = 9.1 × 10^−16^) and ‘positive regulation of cytokine production’ (IRAK3, CEBPB, C3, ADIPOQ, PPARG, IL10, CIDEA; p = 2.1 × 10^−10^). Activation of the triggering receptor expressed on myeloid cells (TREM1) pathway, which amplifies inflammatory signalling, was also seen in response to surgery in omental fat samples (Supplemental Digital Content Fig. 2). This was demonstrated by a positive Z score (2.646, p < 0.0001) on ingenuity pathway analysis (IPA). The Z score in IPA infers the activation state of predicted transcriptional regulators based on experimentally observed gene expression or transcription events.

In VL, there were surprisingly few changes in gene expression in response to surgery, however there was a significant decrease in expression of tripartite motif containing 63 (TRIM 63) gene, also known as muscle ring finger 1 (MURF-1) between pre- and postoperative samples.

#### 3.5.2 Differences between obese and non-obese participants

In postoperative VL samples, gene expression differences indicated increased activation of the peroxisome proliferator-activated receptor (PPAR-α)/retinoid X-receptor (RXR-α) pathway in obese compared with non-obese participants (Supplemental Digital Content Fig. 3). This was demonstrated by a negative Z score (−1.000, p < 0.0001) on IPA. This was confirmed on DAVID analysis which highlighted ‘fatty acid metabolism’ (p = 2.8 × 10^−14^) and PPAR signalling pathway (p = 6.0 × 10^−13^) as the top two enriched pathways from KEGG.

#### 3.5.3 Effects of carbohydrate loading

In VL samples, there were no significant differences in postoperative gene expression between carbohydrate and placebo treated participants. In obese participants, in VL samples, no differences were seen postoperatively between carbohydrate and placebo treatments. There were individual gene expression changes in non-obese carbohydrate loaded participants compared with those receiving placebo but these did not indicate major changes in any particular metabolic or signalling pathway. Postoperatively, in omental fat, there were no differences between samples from carbohydrate-treated or placebo-treated participants. In abdominal fat, there was little influence of carbohydrate treatment or obesity on perioperative changes in gene expression. In particular, carbohydrate loading had no discernible effect in any tissue upon expression of genes involved in carbohydrate metabolism or insulin signalling.

## 4 Discussion

This study has shown that although insulin sensitivity was decreased after major surgery in both obese and non-obese subjects, preoperative carbohydrate treatment or obesity did not have an impact on the magnitude of change. In addition, gene expression analyses suggested that both lipid and carbohydrate metabolism pathways were activated by surgery, but not altered by carbohydrate treatment. Although previous reports suggest increased risk of morbidity in obese patients undergoing surgery [15], in the present study, there were no differences in postoperative complications between obese and non-obese participants nor between carbohydrate and placebo treated participants. The Clavien–Dindo classification [14] is a standard and accepted means of reporting postoperative complications. Although this study was not powered to detect a difference in complication rate, previous meta-analyses have not shown a difference in postoperative complications between carbohydrate and placebo treated individuals [11], [12].

It has been suggested that preoperative carbohydrate treatment reduces the magnitude of postoperative IR [7], [8], [9]. In a small study in colorectal surgery (n = 14), administration of 100 g oral carbohydrate on the night before and 50 g on the morning of surgery was associated with less reduction in whole body glucose disposal compared with the fasted group [7]. There have been two other small studies utilising the HEC which have demonstrated a reduction in IR in preoperative carbohydrate treated patients compared with placebo [8], [9]. In a recent animal study, postoperative whole body glucose disposal rate was higher after carbohydrate treatment than fasting and this correlated negatively with muscle PDK4 protein expression [16]. The same study group demonstrated, in pigs, that a single dose of carbohydrate preoperatively was sufficient to reduce postoperative IR, as measured by the HEC [17]. However, a recent multiple-treatments meta-analysis on 62 participants studied using HEC showed no significant difference in any of the comparisons (between carbohydrate treatment and fasting, water or placebo) [12].

Serum markers of the inflammatory response increased with surgery. There was no significant change in TNF-α but there was a significant change in IL-6. This is in keeping with previous studies of major surgery and suggests that IL-6 may be the main driver of the postoperative response [18] while TNF-α is involved in the inflammatory response to sepsis [19].

Gene expression analysis of abdominal fat, VL and omental fat before and after surgery indicated that adaptive changes in gene expression did occur in response to surgery, but not in response to carbohydrate treatment. Changes in the PPARα/RXRα pathway were demonstrated in postoperative VL samples of obese compared with non-obese participants. PPARα is a master transcriptional regulator of fatty acid oxidation [19], [20], [21], [22] and the PPARα pathway is induced during fasting. In mouse models, PPARα agonists reduced adiposity, decreased muscle and hepatic steatosis and consequently improved insulin sensitivity [23], [24], [25], [26]. Activation of this pathway in the present study may be reflective, in part, of preoperative fasting but this does not account for the increased activation in obese compared with non-obese participants. There are some parallels with the Randle hypothesis which suggested in rats that substrate competition between NEFAs and glucose served as an energy source for muscle [27], resulting in a relative increase in fat oxidation compared with carbohydrate metabolism in response to increased NEFAs.

**Fig. 3:**
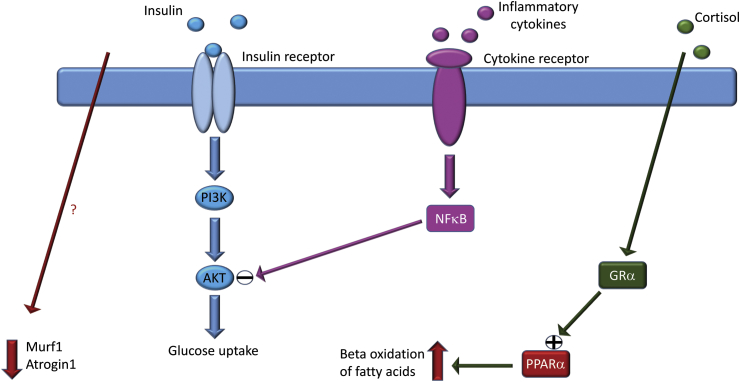
In surgical trauma, increased production and release of inflammatory cytokines leads to NFκB-mediated inhibition of insulin signalling and subsequent decrease in glucose uptake and utilization. Increased glucocorticoid release on the other hand will increase expression of PPARα and lead to an increase in beta oxidation of fatty acids. In concert with these changes in metabolic substrate utilization, the expression of Murf1 and Atrogin1 are decreased post operatively by and as yet unknown mechanism. The net result of these changes re glucose and protein being spared in exchange for increased utilization of fatty acids in the short-term response to surgical trauma. (AKT = protein kinase B, GRα = glucocorticoid receptor α, Murf1 = muscle RING-finger protein-1, NFκB = nuclear factor kappa-light-chain-enhancer of activated B cells, PI3K = phosphatidylinositide 3-kinase, PPARα = peroxisome proliferator-activated receptor α).

Muscle atrophy has been observed in response to protein energy malnutrition and fasting, but is also a feature of immobility, diabetes, cancer cachexia and sepsis [28]. An increased expression of TRIM63 gene (MURF-1) has been observed in models of muscle wasting, including fasting [29]. However, in a study examining skeletal muscle of volunteers subjected to a short-term fast, there was no significant change in MURF-1 expression. Therefore, the changes observed in the present study in TRIM63 and related genes are unlikely to be due to fasting alone [30]. Induction of TRIM63 has been observed in immobilisation-induced muscle atrophy [30] but the length of surgery in the present study is unlikely to have been sufficient to induce atrophy. In this study, TRIM 63 gene expression was decreased postoperatively in VL samples, which is counterintuitive in the context of the current literature. In addition, FBOX032 (atrogin) expression decreased. Atrogin belongs to the ubiquitin proteasome pathway, the primary pathway involved in intracellular protein degradation in skeletal muscle [26]. A decrease in TRIM 63 (MURF 1) and atrogin (FBOX032) would suggest adaptation to counteract myofibril degradation (Fig. 3). Transgenic mice overexpressing TRIM63 did not present with muscle atrophy but had hyperinsulinemia and reduced hepatic glycogen stores [31]. The changes demonstrated in the present study have also been described following stimulation of a catabolic state resulting in muscle atrophy [32].

The most conspicuous finding in omental fat was activation of the TREM1 pathway postoperatively. TREM1 expression has so far been studied primarily in immune cells and its activation resulted in increased production of pro-inflammatory cytokines such as IL-1β and TNFα as well as chemokines such as IL-8 and MCP-1. Surface expression of TREM1 in peripheral blood mononuclear cells was increased significantly in postoperative patients compared with controls, with an even higher expression observed in septic patients [33]. In acute pancreatitis, expression of TREM-1 correlated with disease severity [34]. The activation of the TREM1 pathway in omental samples postoperatively is likely to represent part of the inflammatory response to surgery.

The present study has some limitations. There was heterogeneity in terms of participant age and comorbidities. Every effort was made to ensure that procedures were comparable, in terms of length of procedure and blood loss, but in this relatively small patient group, the variability may have affected results. A standard technique of calculation of insulin dose using body surface area was used [13] but this may have overestimated the insulin dose required per unit of lean mass in obese individuals and, thereby, resulted in greater glucose disposal. However, when the M value was corrected for the circulating insulin concentration during the clamp, there remained no significant difference in the change in perioperative insulin sensitivity between lean and obese individuals.

The HEC was performed with an intentionally high insulin dose to suppress hepatic (and possibly renal) gluconeogenesis and to overcome at least the expected insulin resistance of the obese group. Hence, our findings are not directly comparable with those in the literature using HEC to evaluate insulin sensitivity changes in surgical patients, but the same protocol was used pre- and postoperatively. Thus, the results demonstrate that even at these high rates of insulin infusion, postoperative IR is substantial. The glucose infused during the HEC to maintain euglycaemia is a measure of insulin sensitivity/resistance. In starvation or after trauma/surgery the insulin resistance is reflected by a lower rate of glucose infusion at a fixed insulin infusion. In both conditions, the endocrine and cytokine responses will stimulate hepatic gluconeogenesis, but the insulin infusion rate selected for this study should suppress most if not all of this increased glucose production. This can only be absolutely confirmed with tracer infusions, which were not performed in this study. Thus, it is possible there was still some residual endogenous glucose production, but this will not have offset the almost 50% reduction in glucose infusion rate in the post-surgical state.

In conclusion, the evidence for recommendation of preoperative carbohydrate treatment care is not as strong as believed previously [35] and further large scale well-designed randomised controlled studies looking at patient-centred outcomes are needed before a firm conclusion can be made on its efficacy. It is also possible that due to the overall improvement in perioperative care including avoidance of prolonged preoperative starvation and introduction of other measures to reduce perioperative inflammation, the marginal gains noted in initial studies on carbohydrate treatment may not be apparent in a multimodal pathway designed to decrease the postoperative response to metabolic stress.

## Author contributions

NT – study design, literature search, data collection, data analysis, data interpretation, writing of the manuscript and final approval.
SA – study design, literature search, data interpretation, writing of the manuscript and final approval.
FD – study design, data collection, data interpretation, writing of the manuscript and final approval.
JPW – study design, data interpretation, critical review and final approval.
AB – study design, literature search, data analysis, data interpretation, critical review, supervision and final approval.
IAM – study design, data interpretation, critical review, supervision and final approval
DNL – study design, literature search, data interpretation, figures, writing of the manuscript, critical review, supervision and final approval.

## Funding

This work was supported by the 10.13039/501100000265Medical Research Council [grant number MR/K00414X/1], 10.13039/501100000341Arthritis Research UK [grant number 19891], the CORE Foundation, the European Society for Clinical Nutrition and Metabolism (ESPEN), the National Institute for Health Research Nottingham Digestive Diseases Biomedical Research Unit, and Nottingham University Hospitals Charities.

## Role of funding bodies

The funders had no role in the study design, conduct of the study, data collection or analysis or interpretation, and writing of the paper or the decision to submit for publication. No payment has been received from any other source or agency. The corresponding author has full access to all the data in the study and has final responsibility for the decision to submit for publication.

This paper presents independent research funded by the National Institute for Health Research (NIHR). The views expressed are those of the authors and not necessarily the views of the NHS, the NIHR or the Department of Health.

## Conflict of interest

None of the authors has any direct conflicts of interest to declare. IAM has received research funding from Mars Inc. and serves on the advisory board of IKEA for unrelated work. DNL has received unrestricted research funding and speaker's honoraria from Fresenius Kabi, BBraun and Baxter Healthcare for unrelated work.

## Appendix A Supplementary data

mmc1
