# Supplementary material for: Postoperative inflammation and insulin resistance in relation to body composition, adiposity and carbohydrate treatment: A randomised controlled study
Source: Clin Nutr. 2019 Feb;38(1):204–12. doi: 10.1016/j.clnu.2018.01.032 (PMC6380471; doi:10.1016/j.clnu.2018.01.032)
Supplement: mmc1 [file mmc1.zip › Supplementary digital content.docx]

**SUPPLEMENTAL DIGITAL CONTENT**

##

## EXPERIMENTAL

*Body composition analysis*

Electronic copies of CT scans taken routinely for clinical reasons were obtained from the hospital Picture Archiving and Communication System (PACS). Once accessed, the scans were anonymized, and one CT image slice at the third lumbar vertebrae (L3) level was selected. The images were analysed using SliceOmatic V4.2 software (Tomovision, Montreal, Canada) to calculate the surface area of the specific tissue types: skeletal muscle tissue, visceral adipose tissue and subcutaneous/intramuscular adipose tissue. Within the L3 region are the following muscles: psoas, erector spinae, quadratus lumborum, transversus abdominis, external and internal oblique and rectus abdominis.

SliceOmatic software relies on the variation in density of the different tissue types to identify and thereby quantify the surface area of the tissue present. The different tissue densities are represented by specific Hounsfield Unit (HU) thresholds. The HU thresholds used for skeletal muscle were -29 to +150,^1^ for visceral adipose tissue were -150 to -50^2^ and for subcutaneous and intramuscular adipose were -190 to -30.^3^ Once the tissues were identified, the cross-sectional surface area (cm^2^) of each tissue was calculated automatically by the software.^4^ Any change in tissue area was expressed as absolute change (cm^2^). These data were used to estimate whole body stores of fat-free mass (FFM) and fat mass (FM) using the regression equations of Mourtzakis *et al*:^5^

The cross-sectional area of skeletal muscle was normalized for patient height by calculating the skeletal muscle index (SMI) (cm^2^ m^-2^). Participants were classified as sarcopenic according to established cut offs;^6^ L3 skeletal muscle index (SMI) ≤ 38.5 cm^2^ m^-2^ for women and ≤ 52.4 cm^2^ m^-2^ for men.

In addition, CT scans were analyzed for myosteatosis. The mean HU measurement of all skeletal muscles within the L3 cross-section was recorded as a measure of myosteatosis, which was defined operationally as a mean skeletal muscle radiodensity of <33 HU in those with a BMI ≥25 kg m^-2^ and <41 HU in those with a BMI <25 kg m^-2^ across the axial orthogonal view.^7^

## *Collection and analysis of blood samples*

Serum or plasma was collected in appropriate vacutainers and centrifuged at 4000 *g* for 10 minutes. The serum or plasma was removed and stored in aliquots at -80˚C until analysis. Serum TNFα was measured using a Sandwich enzyme-linked immunosorbent assay (ELISA) method (BD BioSciences, San Diego, California, USA). IL-6 was measured using a Sandwich ELISA method (BD BioSciences). CRP was measured using an immunoturbidimetric assay, TAG using a spectrophotometric assay and glucose using a spectrophotometric assay (all Horiba Medical, Montpelier, France). Cortisol was measured in serum using competitive solid phase ELISA (Alpha Diagnostics International, San Antonio, Texas, USA). Serum insulin was measured using a solid-phase ^125^I radioimmunoassay (Coat-a-count insulin kit, Siemens Medical Solutions Diagnostics, Camberley, UK). Serum NEFA/FFA were measured using an automated immunoassay analyser (Olympus AU5400, Olympus Corporation, Tokyo, Japan) and a commercially available kit (Randox®kit NEFA RB1007, County Antrim, UK).

## *cDNA preparation*

Reverse Transcription

Affinity Script Multiple Temperature Reverse Transcriptase (Agilent Technologies, Santa Clara, CA) was used for reverse transcription from RNA to cDNA, following the standard protocol and manufacturer’s instructions, using random primers (Invitrogen, Paisley, UK). RNAse out Recombinant Ribonuclease Inhibitor (Life Technologies, Paisley, UK) was also added to avoid RNA degradation. 500 ng total RNA was used as a template for synthesis of first strand cDNA.

Real-time quantitative polymerase chain reaction (RT-qPCR)

The template cDNA generated from the reverse transcription reaction was used as the basis for RT-qPCR using TaqMan® Universal PCR Master Mix (Life Technologies, Carlsbad, CA).

*TaqMan low density gene array analysis*

A custom TaqMan® Array Micro Fluidic Card was designed for each tissue (Life Technologies) (Supplementary Tables 2 and 3). This was a 384-well card preloaded with TaqMan® gene expression tests from Life Technologies inventoried assays, in format 96a with 95 target assays and a mandatory reference gene. The reference gene which was automatically included on the card was Homo sapiens glyceraldehyde-3-phosphate dehydrogenase (GAPDH), transcript variant 1 assay reference Hs99999905_m1 (Life Technologies).

**Supplementary Table 1:** Adipose tissue gene card

| **Adipose Tissue Gene Card** | **Expanded gene name/ Protein product** |
| --- | --- |
| ACACA | Acetyl CoA Carboxylase Alpha |
| ALOX15B | Arachidonate 15-lipoxygenase type B |
| ALOX15 | Arachidonate 15-lipoxygenase |
| ATF6 | Activating Transcription Factor 6 |
| ATG5 | Autophagy Related Gene 5 |
| ATG7 | Autophagy Related Gene 7 |
| BCL2 | B-cell lymphoma 2 |
| BECN1 | Beclin 1 |
| C3 | Complement component 3 |
| CASP1 | Caspase 1 |
| **GAPDH** | **Glyceraldehyde 3-phosphate dehydrogenase** |
| CASP3 | Caspase 3 |
| CASP7 | Caspase 7 |
| CD36 | Cluster of differentiation 36 |
| CEBPA | CCAAT enhancer binding protein alpha |
| CEBPB | CCAAT enhancer binding protein beta |
| CES1 | Carboxylesterase 1 |
| CLEC7A | C-type lectin domain family 7 |
| CRAT | Carnitine acetyltransferase |
| DDIT3 | DNA damage inducible transcript 3 |
| DERL2 | Degradation in endoplasmic reticulum 2 |
| DLK1 | Delta-like 1 homolog |
| EDEM2 | ER degradation enhancer, mannosidase alpha-like 1 |
| EPHX2 | Epoxide hydrolase 2 |
| FAS | TNF receptor superfamily member 6 |
| FOXO1 | Forkhead box 01 |
| HIF1A | Hypoxia inducible factor 1A |
| IGFBP2 | Insulin-like growth factor binding protein 2 |
| IL10 | Interleukin 10 |
| IL1B | Interleukin 1B |
| IL18 | Interleukin 18 |
| IL6 | Interleukin 6 |
| IL8 | Interleukin 8 |
| IRAK1 | Interleukin receptor associated kinase 1 |
| IRAK3 | Interleukin receptor associated kinase 3 |
| LCN2 | Lipocalin 2 |
| LPCAT1 | Lysophosphatidylcholine acyltransferase 1 |
| LPCAT2 | Lysophosphatidylcholine acyltransferase 2 |
| MAP1LC3A | Microtubule associated protein 1 light chain 3 alpha |
| MAP1LC3B | Microtubule associated protein 1 light chain 3 beta |
| MMP12 | Matrix metallopeptidase 12 |
| MMP3 | Matrix metallopeptidase 3 |
| MYD88 | Myeloid differentiation primary response 88 |
| NAMPT | Nicotinamide phosphoribosyltransferase |
| NFE2L2 | Nuclear factor (erythroid-2-like-2) |
| NLRP3 | NLR family, pyrin domain containing 3 |
| PDK1 | Pyruvate dehydrogenase kinase 1 |
| PNPLA2 | Patatin like phospholipase domain containing 2 |
| PNPLA3 | Patatin like phospholipase domain containing 3 |
| PPARD | Peroxisome proliferator activated receptor delta |
| SOCS3 | Suppressor of cytokine signalling 3 |
| SOD1 | Superoxide dismutase 1 |
| SOD2 | Superoxide dismutase 2 |
| SREBF1 | Sterol regulatory element binding transcription factor 1 |
| TGFB1 | Transforming growth factor beta 1 |
| TIMP1 | TIMP metallopeptidase inhibitor 1 |
| TNF | Tumour necrosis factor |
| TNFAIP6 | Tumour necrosis factor, alpha induced protein 6 |
| ADIG | Adipogenin |
| ADIPOQ | Adioponectin c1Q and collagen domain containing |
| CCL2 | Chemokine (C-C motif) ligand 2 |
| CD68 | Cluster of differentiation 68 |
| CEBPD | CCAAT enhancer binding protein delta |
| CFD | Complement factor d |
| CIDEA | Cell death inducing DFFA-like effector a |
| FABP4 | Fatty acid binding protein 4 |
| FAM101A | Family with sequence similarity 101, member A |
| IRS1 | Insulin receptor substrate 1 |
| IRS2 | Insulin receptor substrate 2 |
| IRS4 | Insulin receptor substrate 4 |
| ITGAX | Integrin, alpha X |
| LEP | Leptin |
| LPIN1 | Lipin 1 |
| LPL | Lipoprotein lipase |
| MTX1 | Metaxin 1 |
| PLIN1 | Perilipin 1 |
| PPARG | Peroxisome proliferator activated receptor gamma |
| PPARGC1A | Peroxisome proliferator activated receptor gamma, co-activator 1 alpha |
| RETN | Resistin |
| RPUSD3 | RNA pseudouridylate synthase domain containing 3 |
| SLC2A4 | Solute carrier family 2, member 4 |
| CD3E | Epsilon |
| GATA3 | GATA binding protein 3 |
| GSTK1 | Glutathione S-transferase kappa 1 |
| HERPUD1 | Homocysteine-inducible endoplasmic reticulum stress inducible, ubiquitin-like domain receptor 1 |
| HP | Haptoglobin |
| LIPE | Hormone sensitive lipase |
| **RORC** | **RAR-related orphan receptor C** |
| TBX21 | T-box 21 |
| **TBP** | **TATA box binding protein** |
| BLANK |  |
| TRPV4 | Transient receptor potential cation channel, subfamily V member 4 |
| BLANK |  |
| **ACTB** | **Beta-actin** |
| BLANK |  |
| HMBS | Hydroxymethylbilane synthase |

Housekeeping genes are highlighted in bold font

**Supplementary Table 2:** Skeletal muscle gene card

| **Skeletal Muscle Gene Card** | **Expanded gene name/ Protein product** |
| --- | --- |
| GRB14 | Growth factor receptor bound protein 1 |
| **GPD2** | **Glycerol-3-phosphate dehydrogenase 2** |
| FGF21 | Fibroblast growth factor 21 |
| FOXO3 | Forkhead box 03 |
| SCD | Stearoyl Co A Desaturase |
| ANGPTL4 | Angiopoietin-like 4 |
| FASN | Fatty acid synthase |
| NRF1 | Nuclear respiratory factor 1 |
| NUAK2 | NUAK family, SNF-1 like kinase |
| LCP1 | Lymphocytic cytosolic protein 1 |
| FSTL1 | Follistatin-like 1 |
| G6PD | Glucose 6 phosphate dehydrogenase |
| ERN1 | Endoplasmic reticulum to nucleus signalling 1 |
| SCD5 | Stearoyl Co A Desaturase 5 |
| ACLY | ATP citrate lyase |
| GREM1 | Gremlin 1 |
| NR4A3 | Nuclear receptor subfamily 4, group A, member 3 |
| NR4A1 | Nuclear receptor subfamily 1, group A, member 1 |
| IGF1 | Insulin-like growth factor 1 |
| MYEF2 | Myelin expression factor 2 |
| CD36 | Thrombospondin receptor |
| CPT1B | Carnitine palmitoyltransferase 1B |
| SLC2A1 | Solute carrier family 2 member 1 |
| ATF3 | Activating transcription factor 3 |
| ME1 | Malic enzyme 1 |
| **RPLP2** | **Ribosomal protein P2** |
| PRKCE | Protein kinase C, epsilon |
| PPARGC1B | Peroxisome proliferator activated receptor gamma, coactivator 1 beta |
| TDO2 | Tryptophan 2-3, dioxygenase |
| IL6 | Interleukin 6 |
| UCP3 | Uncoupling protein 3 |
| ABL1 | Abelson murine leukaemia 1 |
| ACACA | Acetyl CoA Carboxylase Alpha |
| RRAD | Ras related associated with diabetes |
| PKM | Pyruvate kinase, muscle |
| MLXIPL | MLX interacting protein like |
| CASP3 | Caspase 3 |
| ACADL | Acyl CoA dehydrogenase |
| ACADVL | Acyl CoA dehydrogenase, very long chain |
| PDK2 | Pyruvate dehydrogenase kinase 2 |
| SLC7A11 | Solute carrier family 7 (anionic amino acid transporter y+ system), member 11 |
| PPARA | Peroxisome proliferated activated receptor alpha |
| SLC2A4 | Solute carrier family 2 (facilitated glucose transporter), member 4 |
| LCN2 | Lipocalin |
| FOXO1 | Forkhead box 01 |
| IL8 | Interleukin 8 |
| ARRDC4 | Arrestin domain containing 4 |
| KSR2 | Kinase suppressor of ras 2 |
| TAT | Tyrosine aminotransferase |
| ELOVL6 | Fatty acid elongase 6 |
| DDIT4 | DNA damage inducible transcript 4 |
| NDUFA7 | NADH dehydrogenase alpha subcomplex subunit 7 |
| PRKAA1 | Protein kinase AMP activated alpha 1 catalytic subunit |
| **TBP** | **TATA box binding protein** |
| FABP3 | Fatty acid binding protein 3 |
| PPARGC1A | Peroxisome proliferated activated receptor gamma, coactivator 1 alpha |
| TRIM63 | Tripartite motif containing 63 |
| IRS2 | Insulin receptor substrate 2 |
| ACACB | Acetyl CoA Carboxylase Beta |
| CEBPB | CCAAT enhancer binding protein B |
| KLF10 | Kruppel-like factor 10 |
| PDK4 | Pyruvate dehydrogenase kinase 4 |
| MLYCD | Malonyl Ca O decarboxylase |
| DDIT3 | DNA damage inducible transcript 3 |
| PPARD | Peroxisome proliferated activated receptor delta |
| IL15 | Interleukin 15 |
| ERO1L | Endoplasmic reticulum oxidoreductase –like |
| **HPRT1** | **Hypoxanthine phosphoribosyltransferase 1** |
| HSPA5 | Heat shock 70kDa protein 5 |
| UCP2 | Uncoupling protein 2 |
| SREBF1 | Sterol regulatory binding transcription factor 1 |
| GAPDH | Glyceraldehyde 3 phosphate dehydrogenase |
| CHI3L1 | Chitonase 3 like 1 |
| MYOG | Myogenin |
| IRS1 | Insulin receptor substrate 1 |
| EGR1 | Early growth response 1 |
| TRIB3 | Tribbles homolog 1 |
| TNF | Tumour necrosis factor |
| STAT3 | Signal transducer and activator of transcription |
| CRTC2 | CREB regulated transcription coactivator 2 |
| BHLHE40 | Basic loop helix family member E40 |
| IL6R | Interleukin 6 receptor |
| HK2 | Hexokinase 2 |
| CARM1 | Coactivator associated methyltransferase 1 |
| HAS2 | Hyaluronan synthase 2 |
| TXNIP | Thioredoxin interacting protein |
| PPP1R3A | Protein phosphatase 1 regulatory subunit 3A |
| SREBF2 | Sterol regulatory binding transcription factor 1 |
| ACSL4 | Acyl CoA Synthetase long chain family member 4 |
| RASD1 | Ras, dexamethasone induced 1 |
| SIRT1 | Sirtuin 1 |
| FBX032 | F-Box protein 32/ Atrogin |
| ATF4 | Activating transcription factor 4 |
| SOCS3 | Suppressor of cytokine signalling 3 |
| TFE3 | Transcription factor binding to IGHM enhancer 3 |
| GSTA5 | Glutathione S transferase alpha 5 |

Housekeeping genes are highlighted in bold font

A 7900HT Fast Real-Time PCR System (Life Technologies) was used following the standard protocol (Applied Biosystems Part Number 4400263). 110 ng cDNA (from total RNA) was used per lane, in duplicate for each sample. TaqMan® Universal PCR Master Mix (Life Technologies) was used as recommended.

Target genes were selected from background research and this list extended using IPA interactive pathways analysis software (Ingenuity Systems, Redwood City, CA).

Quantification

The comparative CT method was used to compare the gene expression in two different samples (sample A and sample B); with each sample related to a geometric mean of housekeeping genes prior to comparison. The three housekeepers were checked for normal distribution and correlation to each other. Target gene delta-CTs were also checked for normal distribution. Fold change was calculated as described previously, using 2^-delta CT^.^8,9^

Interpretation of comparative results

As expression levels were already normalised against control genes prior to analyses their results could be negative or positive, dependent on the expression differential to the control. CT values that are larger indicate lower expression and later amplification, and lower CT values indicate higher expression and earlier amplification. ∆CT expression levels that are negative indicate expression of the transcript above that of the control and positive values indicate expression levels below that of the control. Therefore ∆CT values that are negative indicate expression levels higher than positive values. When correlations or association analyses are carried out, the direction of association has to take into account the inverse relationship between expression level and CT value.

**RESULTS**

**Supplementary Table 3:** Surgical procedures performed (All open, no laparoscopic procedures)

| **Surgery type** | **Total** | **Obese** | **Non-obese** |
| --- | --- | --- | --- |
| **Major pancreaticobiliary surgery**  Carbohydrate  No carbohydrate | 17  8  9 | 7  3  4 | 10  5  5 |
| **Major/complex colorectal surgery**  Carbohydrate  No carbohydrate | 12  5  7 | 7  3  4 | 5  2  3 |
| **Abdominal wall reconstruction**  Carbohydrate  No carbohydrate | 3  3  0 | 2  2  0 | 1  1  0 |

Supplementary Table 4: Results of different methods of body composition analysis

| **Body composition measures according to groups** | **DXA measurement (Mean**±**SD) (kg)** | **CT measurement (Mean**±**SD) (kg)** |
| --- | --- | --- |
| **Overall**  Fat mass  Lean mass/ Fat free mass | 27.14 ± 7.93  48.52 ± 10.26 | 26.52 ± 7.69  54.80 ± 11.78 |
| **Obese**  Fat mass  Lean mass/ Fat free mass | 31.38 ± 6.43  51.70 ± 10.72 | 30.51 ± 5.75  60.89 ± 12.54 |
| **Non-obese**  Fat mass  Lean mass/ Fat free mass | 23.17 ± 7.23  45.54 ± 9.16 | 22.82 ± 4.12  49.14 ± 7.80 |
| **Difference between obese and non-obese (p value)**  Fat mass  Lean mass/ Fat free mass | **0.002**  0.096 | **0.007**  **0.007** |

**Supplementary Figure 1:** Insulin concentrations during hyperinsulinaemic euglycaemic clamp


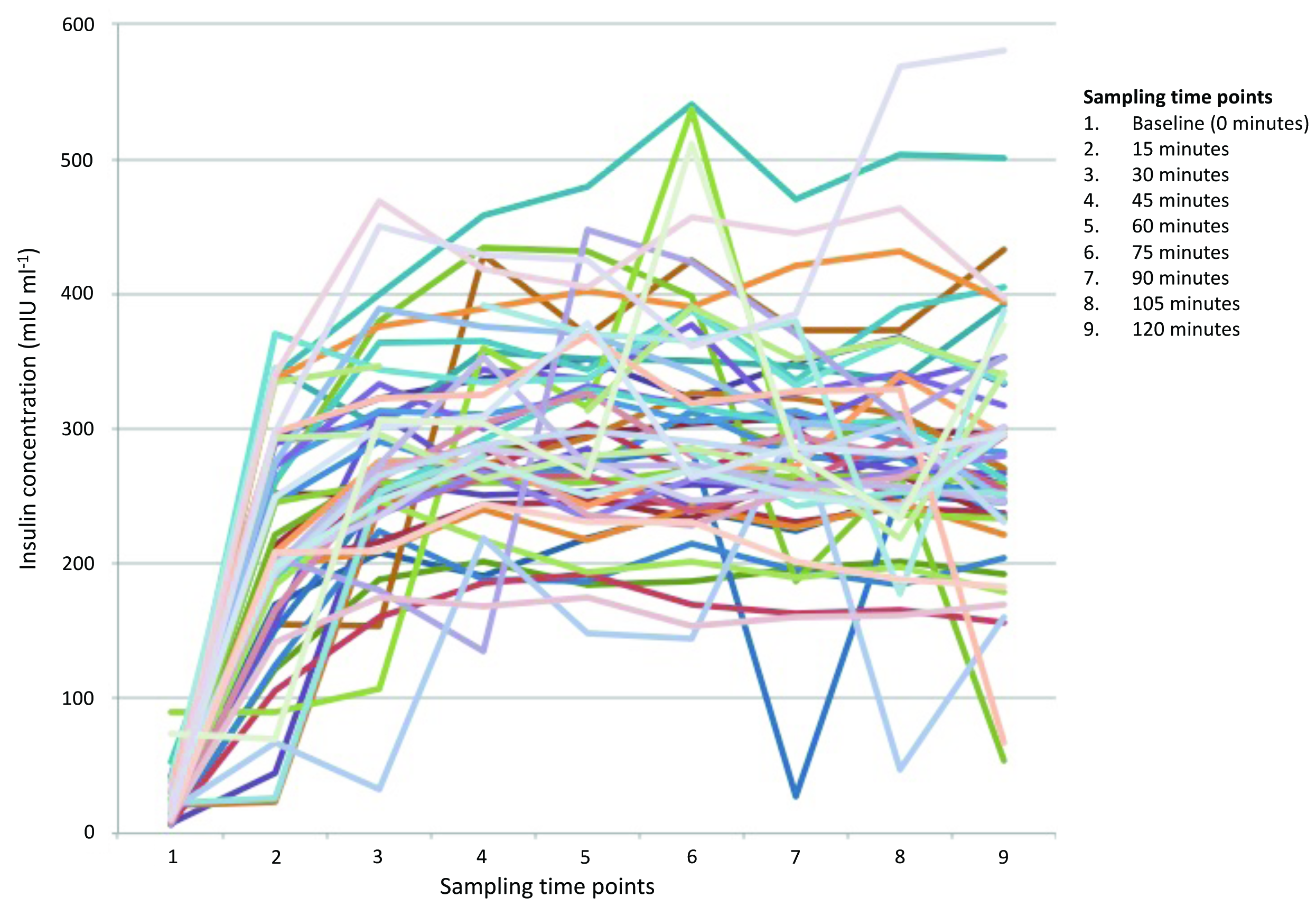


**Supplementary Figure 2:** TREM-1 is a member of the Ig superfamily expressed on circulating and tissue resident immune cells. It is a 30-kDa glycoprotein receptor containing a short intracytoplasmic domain which does not contain any signaling motifs. TREM1 must associate with a signaling unit DAP12, to induce intracellular pathways Association of TREM-1 with DAP12 activates intracellular pathways, directly inducing production of inflammatory cytokines such as IL-6 and IL-8. Expression of these cytokines subsequently leads to a further increase in TREM1 expression and activity. Increased cytokine production will lead to temporary insulin resistance in metabolically active tissues.


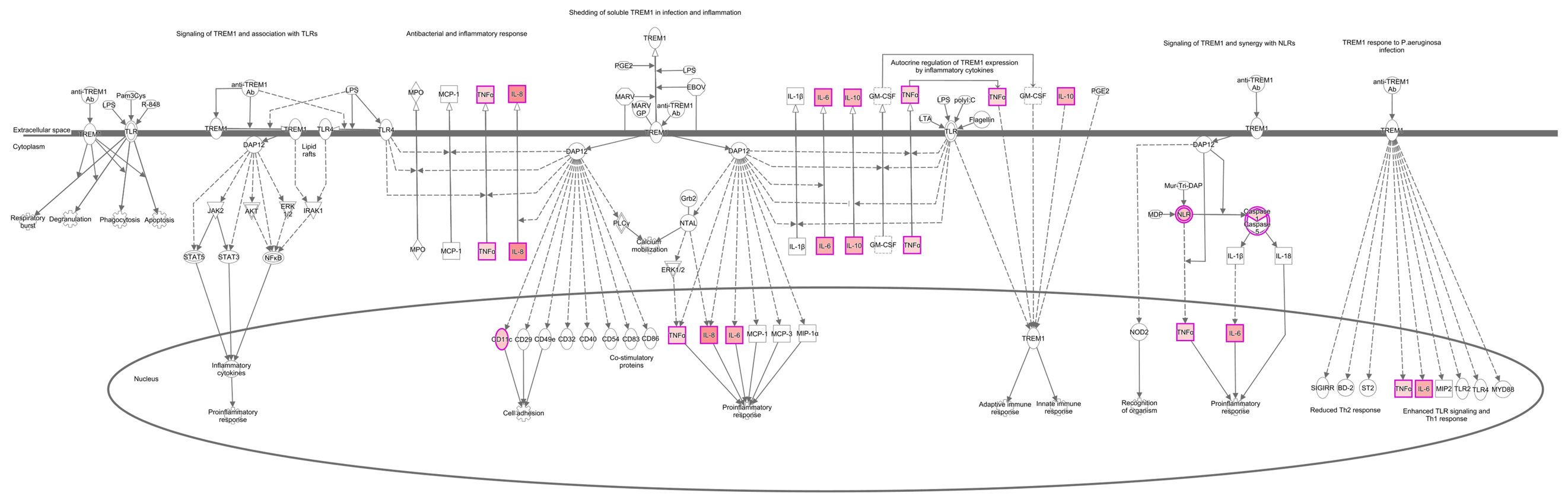


**Supplementary Figure 3:** PPARα is a ligand activated nuclear receptor that acts as a heterodimer with RXR to induce transcription of genes involved in glucose and lipid metabolism. PPARα expression can be induced by glucocorticoids which will lead to increased expression of beta oxidation genes such as Acyl CoA Oxidase and Long chain CoA Acetyl Transferase in metabolically active tissues. This will lead to an increase in fatty acid utilization which will compensate for the reduced glucose utilization post-surgery.


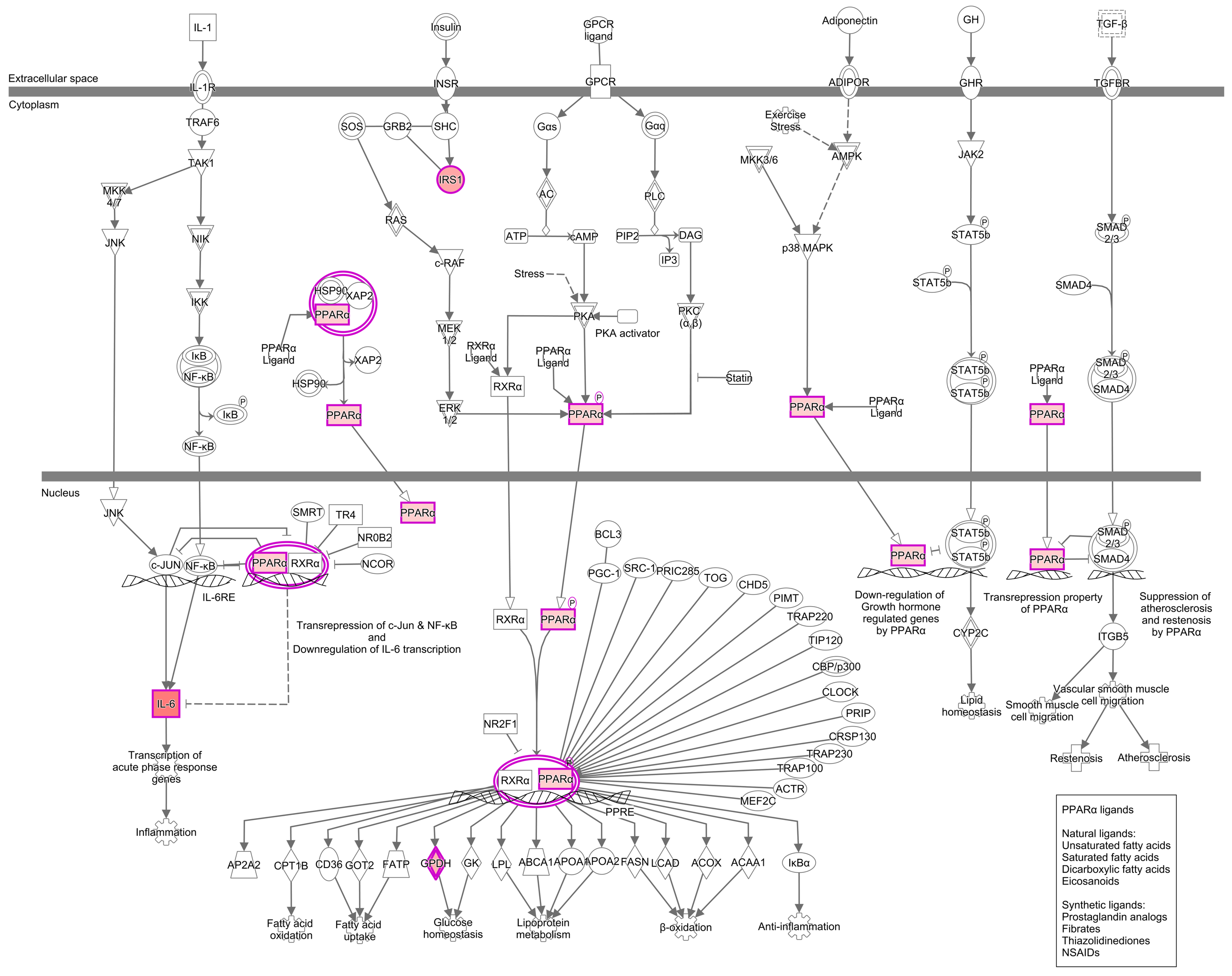


**REFERENCES**

1. Mitsiopoulos N, Baumgartner RN, Heymsfield SB, et al: Cadaver validation of skeletal muscle measurement by magnetic resonance imaging and computerized tomography. *J Appl Physiol* 1998; 85(1): 115-122

2. Vehmas T, Kairemo KJA, Taavitsainen MJ: Measuring visceral adipose tissue content from contrast enhanced computed tomography. *Int J Obes (Lond)* 1996; 20(6): 570-573

3. Kvist H, Sjostrom L, Tylen U: Adipose-tissue volume determinations in women by computed-tomography - technical considerations. *Int J Obes (Lond)* 1986; 10(1): 53-67

4. Prado CM, Baracos VE, McCargar LJ, et al: Body composition as an independent determinant of 5-fluorouracil-based chemotherapy toxicity. *Clin Cancer Res* 2007; 13(11): 3264-3268

5. Mourtzakis M, Prado CMM, Lieffers JR, et al: A practical and precise approach to quantification of body composition in cancer patients using computed tomography images acquired during routine care. *Appl Physiol Nutr Metab* 2008; 33(5): 997-1006

6. Prado CM, Lieffers JR, McCargar LJ, et al: Prevalence and clinical implications of sarcopenic obesity in patients with solid tumours of the respiratory and gastrointestinal tracts: a population-based study. *Lancet Oncol* 2008; 9(7): 629-635

7. Martin L, Birdsell L, Macdonald N, et al: Cancer cachexia in the age of obesity: skeletal muscle depletion is a powerful prognostic factor, independent of body mass index. *J Clin Oncol* 2013; 31(12): 1539-1547

8. Livak KJ, Schmittgen TD: Analysis of relative gene expression data using real-time quantitative PCR and the 2−ΔΔCT method. *Methods* 2001; 25(4): 402-408

9. Schmittgen TD, Livak KJ: Analyzing real-time PCR data by the comparative CT method. *Nat Protocols* 2008; 3(6): 1101-1108
